# Uncovering the Protective Neurologic Mechanisms of Hypofractionated FLASH Radiotherapy

**DOI:** 10.1158/2767-9764.CRC-23-0117

**Published:** 2023-04-27

**Authors:** Yasaman Alaghband, Barrett D. Allen, Eniko A. Kramár, Richard Zhang, Olivia G.G. Drayson, Ning Ru, Benoit Petit, Aymeric Almeida, Ngoc-Lien Doan, Marcelo A. Wood, Janet E. Baulch, Paola Ballesteros-Zebadua, Marie-Catherine Vozenin, Charles L. Limoli

**Affiliations:** 1 Department of Radiation Oncology, University of California, Irvine, California.; 2 Department of Neurobiology and Behavior, University of California, Irvine, California.; 3 Laboratory of Radiation Oncology, Department of Radiation Oncology, Lausanne University Hospital and University of Lausanne, Lausanne, Switzerland.; 4 Instituto Nacional de Neurología y Neurocirugía MVS, México City, México.

## Abstract

Implementation of ultra-high dose-rate FLASH radiotherapy (FLASH-RT) is rapidly gaining traction as a unique cancer treatment modality able to dramatically minimize normal tissue toxicity while maintaining antitumor efficacy compared with standard-of-care radiotherapy at conventional dose rate (CONV-RT). The resultant improvements in the therapeutic index have sparked intense investigations in pursuit of the underlying mechanisms. As a preamble to clinical translation, we exposed non–tumor-bearing male and female mice to hypofractionated (3 × 10 Gy) whole brain FLASH- and CONV-RT to evaluate differential neurologic responses using a comprehensive panel of functional and molecular outcomes over a 6-month follow-up. In each instance, extensive and rigorous behavioral testing showed FLASH-RT to preserve cognitive indices of learning and memory that corresponded to a similar protection of synaptic plasticity as measured by long-term potentiation (LTP). These beneficial functional outcomes were not found after CONV-RT and were linked to a preservation of synaptic integrity at the molecular (synaptophysin) level and to reductions in neuroinflammation (CD68^+^ microglia) throughout specific brain regions known to be engaged by our selected cognitive tasks (hippocampus, medial prefrontal cortex). Ultrastructural changes in presynaptic/postsynaptic bouton (Bassoon/Homer-1 puncta) within these same regions of the brain were not found to differ in response to dose rate. With this clinically relevant dosing regimen, we provide a mechanistic blueprint from synapse to cognition detailing how FLASH-RT reduces normal tissue complications in the irradiated brain.

**Significance:**

Functional preservation of cognition and LTP after hypofractionated FLASH-RT are linked to a protection of synaptic integrity and a reduction in neuroinflammation over protracted after irradiation times.

## Introduction

Standard radiotherapy at conventional dose rate (CONV-RT) is routinely used to control malignant growth, typically involving photon modalities delivered at mean dose rates in the range of approximately 0.03 Gy/second. Improvements in conformality and stereotactic approaches have greatly improved certain patient outcomes; however, curative intent is still hampered by radioresistant tumor recurrence and resultant normal tissue toxicities that define dose tolerances. For decades, these fundamental limitations have been tackled by tailoring fractionation schedules combined with technological improvements in imaging and beam delivery to squeeze out relatively incremental gains in the therapeutic index. Ultra-high dose-rate FLASH radiotherapy (FLASH-RT) has been shown to afford marked normal tissue sparing while maintaining antitumor efficacy, *in vivo* outcomes that define the “FLASH effect” (1–4).

Recent work has substantiated the broad ranging capability of FLASH-RT using electrons, photons, and protons to alleviate normal tissue toxicities in the brain, lung, gut, blood, bone, muscle, and skin without compromising the tumoricidal activity of ionizing radiation (5). Importantly, these findings regarding normal tissue sparing have been validated in fish, mice, cats, dogs, and mini-pigs as well as in multiple preclinical mouse tumor models (5). The global scope of these far-reaching benefits coupled with the diversity of the normal tissues and tumor types involved has in large part, confounded efforts aimed at elucidating a unifying mechanistic hypothesis able to account for the FLASH effect. Notwithstanding, extensive data derived from the normal mouse brain and mice bearing orthotopic brain tumors have substantiated the promise of FLASH-RT at ameliorating many of the long-lasting neurocognitive and cerebrovascular complications caused by cranial radiotherapy that severely compromise the quality of life of adult and pediatric brain tumor survivors (6–11). In this light, the focus of this study was to advance our mechanistic understanding of how FLASH-RT forestalls (if not eliminates) the progressive onset of the neurologic decrements observed routinely with CONV-RT. The temporal development of normal tissue toxicities associated with standard-of-care cranial radiotherapy for glioblastoma (GBM, 30fx at 2 Gy ± temozolomide) have been well documented (12). Despite promising trials implementing the N-methyl-D-aspartate receptor (NMDAR) antagonist memantine and/or hippocampal sparing (13, 14), multifaceted cognitive deficits are inadequately resolved, in part, because these functional outcomes are readouts of network level disruptions not restricted to perturbations of NMDAR signaling or damage to the temporal lobes.

The foregoing provides the rationale for pursing the mechanistic basis of the FLASH sparing effect in the brains of adult male and female mice exposed to hypofractionated cranial FLASH- and CONV-RT previously validated to control GBM and spare cognition (8). Mice were evaluated on a rigorous behavioral platform beginning 10 weeks after exposure, designed to discriminate the extent of radiation-induced cognitive deficits between the cohorts. In each instance and regardless of sex, FLASH and control cohorts were statistically indistinguishable, whereas CONV cohorts exhibited significant learning and memory impairments on each of the behavioral paradigms administered (Objects in Updated Locations, OUL; Novel Object Recognition, NOR; Light/Dark Box, LDB; and Fear Extinction, FE). Cognitive deficits coincided with impaired synaptic plasticity, as electrophysiological assessments of LTP conducted in the hippocampus and/or medial prefrontal cortex showed that CONV-RT inhibited LTP significantly, whereas FLASH-RT did not. Sparing of these critical functional outcomes in the FLASH irradiated brain was investigated at the molecular and structural levels of the synapse. Results show that FLASH preserved synaptic density (synaptophysin), whereas the structural integrity of presynaptic and postsynaptic bouton (Bassoon/Homer-1) remained unchanged 6 months after radiotherapy. The beneficial neurobiologic effects of FLASH-RT extended to microglia, where a significant increase in the levels of reactive CD68^+^ microglia found after CONV-RT were not evident in FLASH irradiated brains, confirming the relative absence of this key marker of neuroinflammation. Collectively, these new data highlight structural, molecular, and functional endpoints that link the neurologic benefits of FLASH-RT from synapse to cognition.

## Materials and Methods

### Animals

Animal procedures were conducted in accordance with the Swiss ethics committee (VD3603) and the University of California, Irvine Institutional Animal Care and Use Committee (AUP-21-025) for animal experimentation. Male and female C57Bl/6J mice (*n* = 16/treatment/sex) were purchased from Charles River Laboratories (strain code 632) and were allowed to acclimate. Mice were 10 weeks of age at the time of irradiation.

### Irradiation

Whole-brain irradiations were performed on a prototype Oriatron 6e, 6-MeV electron beam linear accelerator (LINAC) at the Lausanne University Hospital (Lausanne, Switzerland), as described previously (15). Extensive description of this prototype Oriatron dosimetry has been described previously (16, 17). Mice received three whole-brain, head only, doses of 10 Gy, separated by 48 hours using a 17-mm graphite applicator at either CONV dose rate (0.09 Gy/second) or ultra-high dose-rate FLASH delivered in a single 1.8 μs pulse (5.6 × 10^6^ Gy/second). Details of the irradiation parameters are listed in Table 1.

**TABLE 1:** Irradiation parameters. Fractionated whole brain irradiation was performed on the Oriatron 6e, 6-MeV electron beam LINAC at Lausanne University Hospital. Mice received three doses of 10 Gy separated by 48 hours using a 17 mm graphite applicator at either CONV (CONV-RT; 0.09 Gy/second) or ultra-high dose-rate FLASH (FLASH-RT, delivered at 5.6 × 10^6^ Gy/second in a single 1.8 μs pulse)

	Prescribed dose and regimen	
	CONV	FLASH
Beam parameters	3 × 10 Gy	3 × 10 Gy
Graphite applicator type and size (mm)	Circular Ø17	Circular Ø17
Source-to-surface distance (mm)	800	209
Pulse repetition frequency (Hz)	10	100
Pulse width	1.0 μsec	1.8 μsec
No. of pulses	1170–1180	1
Treatment time (seconds)	117	1.8 × 10^−6^
Mean dose rate (Gy/second)	0.1	5.6 × 10^6^
Instantaneous dose rate (Gy/second)	8.5 × 10^3^	5.6 × 10^6^

### Experimental Design

**FIGURE 1:**
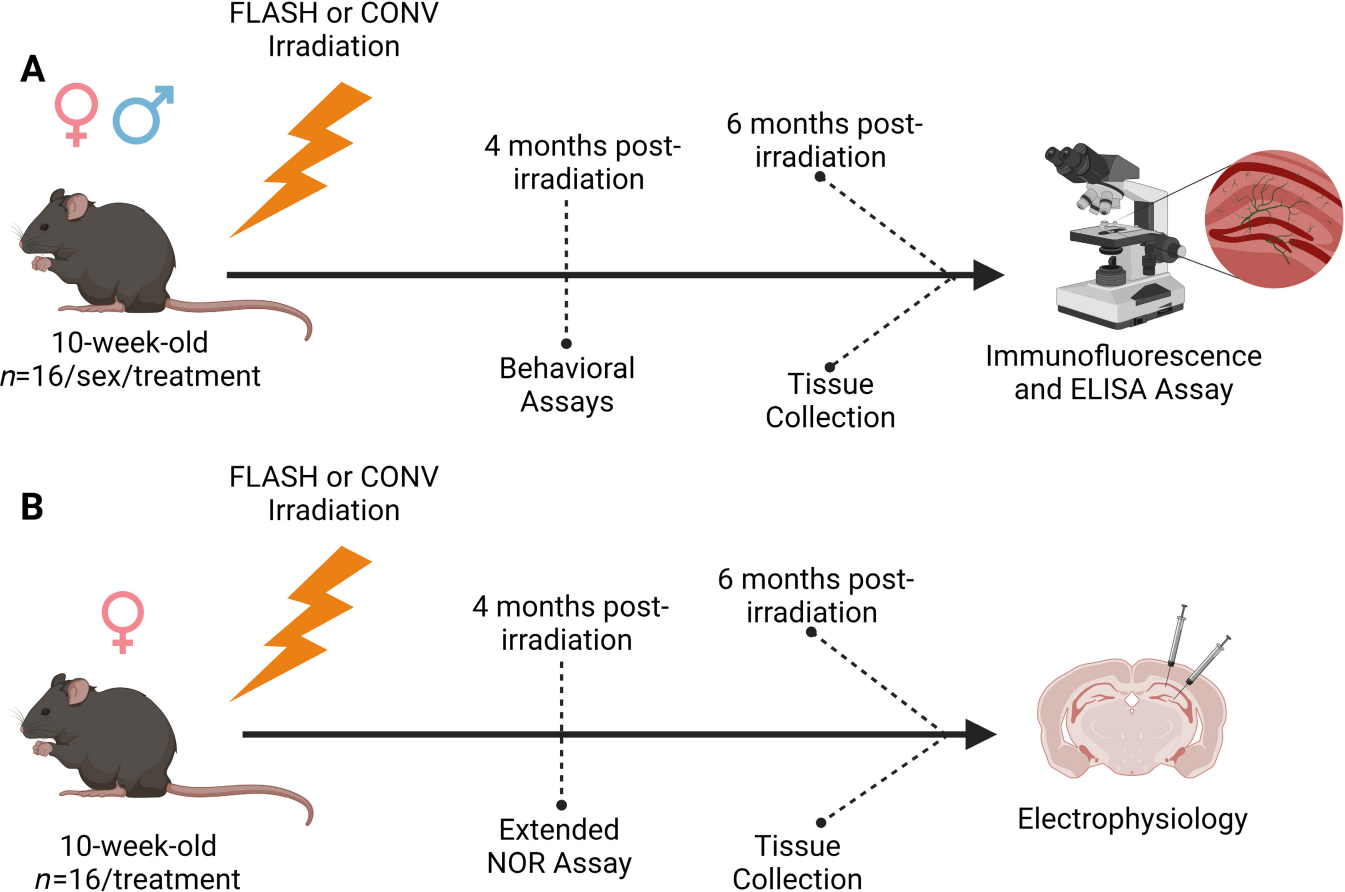
Timeline. Ten-week-old mice received hypofractionated FLASH or CONV irradiation (3 × 10 Gy). **A,** Male and female mice underwent behavioral testing 4 months after irradiation. At 6 months post-irradiation, mice were sampled, and tissues were prepared for either IHC analysis (*n* = 4/sex/treatment) or ELISA (*n* = 5–8/sex/treatment). **B,** Female mice were used to assess LTP. At 4 months post-irradiation, animals performed in the extended NOR behavioral assay (*n* = 16/treatment). At 6 months post-irradiation, mice were sacrificed and prepared for electrophysiological analysis of long-term potentiation (*n* = 10–11/treatment).

To determine the neuromechanistic basis of the FLASH effect, we exposed adult (10-week-old) male and female mice to a hypofractionated dose (3 × 10 Gy, 48 hours apart) of either FLASH or CONV radiotherapy. Four months after irradiation, mice performed in a series of behavior assays to assess radiation-associated cognitive damage. After 6 months, mice were sacrificed, and tissues removed/prepared for assessment of endpoints listed below. Visual representation of the experimental design is presented in Fig. 1. Prior to sacrifice, mice were randomly assigned to either IHC (*n* = 4/treatment/sex), molecular (*n* = 5–8 males/treatment; Fig. 1A) or for electrophysiology (*n* = 10–11 females/treatment; Fig. 1B). Mice designated for IHC analysis were intracardially perfused using 25 mL of heparinized saline followed immediately by 4% paraformaldehyde. Preparation for electrophysiology is described below.

### Behavioral Testing

#### Behavior Apparatus

All behavior was conducted in a dimly lit room inside an arena (30 × 30 × 30 cm) lined with a layer of fresh corncob bedding. During OUL testing, a thin blue strip of duct tape was placed on one of the walls of the arena, to serve as an orientating mark. All plastic toys used for OUL and NOR were cleaned prior to testing. Sessions were recorded offline using IC Capture for the purpose of offline exploration analysis via an overhead camera.

#### OUL Test

Mice were handled for 2 minutes per day for a period of 4 days prior to a period of habituation inside the empty arenas lasting 6 consecutive days. After habituation, mice were trained with two identical plastic toys in specific locations (A1, A2) for 3 days. Toys were magnetically fixed 16 cm apart from one another, and the mice were allowed to explore the context for 5 minutes. Twenty four hours later, one toy was moved to an updated location (A3), and mice were allowed to explore for 5 minutes. Finally, mice were given a retention test, where identical toys were placed in all three previous locations [initial and updated locations (A1, A2, A3)], as well as a fourth toy in a novel location (A4; *n* = 16/sex/treatment). Preference for the various locations was calculated as a discrimination index (DI), [(novel/total exploration time) − (familiar/total exploration time)] × 100.

#### NOR

Mice were habituated for 5 minutes in empty plastic arenas for 1-day after OUL testing. Twenty four hours later, mice were then allowed to explore the arena for 5 minutes with identical plastic toys. Mice were removed from the arena and were placed back into their home cage for 5 minutes. Mice were returned to the arena containing one original and one novel toy and allowed to explore for 5 minutes. Minutes 1–3 were used in the analysis to allow animals a chance to habituate to the toys and arenas (*n* = 13–16/sex/treatment). In addition, female mice used for electrophysiology performed the long-term NOR test which extended the period of time between the training and testing phase from 5 minutes to 24 hours (*n* = 15–16/treatment).

#### LDB

Anxiety behavior was evaluated using the LDB test. The LDB arena comprised of an exposed light section (30 × 20 × 27 cm) connected to a covered dark section (15 × 10 × 27 cm) via a small opening. Mice were allowed to explore the arena for 5 minutes; amount of time spent in each section as well as the number of transitions between the two sections were recorded (*n* = 16/treatment).

#### FE

To test the impact of the treatments on the ability of the mice to learn and extinguish fear responses, we conducted a series of FE experiments. Testing occurred in two similar yet different contexts within a behavioral testing chamber (17.5 × 17.5 × 18 cm, Coulbourn Instruments) consisting of a steel slat floor (3.2 mm diameter slats, 8 mm spacing). In context A, mice were exposed to a vinegar scent, comprised of a solution of 10% vinegar sprayed within the chamber. In context B, metal slats were covered with a plastic tile and mice were exposed to a new scent comprised of a solution of 10% almond extract in a chamber equipped with modified lighting. Fear conditioning was performed in context A, where mice were habituated for 2 minutes; three pairings of a tone (16 kHz, 80 dB, 120 seconds) with a foot shock (0.6 mA, 1 second) were applied to the mice. For the following 3 days, the mice underwent extinction training in context B. Mice underwent 2 minutes of habituation, before a series of 20 unpaired tones (16 kHz, 80 dB, 120 seconds) was applied to the mice. Freezing behavior was recorded by an overhead camera and analyzed using an automated motion detection program (FreezeFrame, Cobourn Instruments). Tones 2 to 12 were used for extinction training analysis to allow animals a brief habituation to the chamber and reduce false freezing behavior when animals stop near the end of the 45 minute long trial (*n* = 16/sex/treatment).

Mice were then finally tested again in context B with 2 minutes of habituation, followed by a series of three unpaired tones (16 kHz, 80 dB, 120 seconds). Data were recorded using the overhead camera setup and freezing behavior was analyzed using FreezeFrame. A threshold separating values for freezing behavior and motion was set by an investigator, based on identifying a trough separating low and high mobility behaviors.

### Postmortem Analysis

#### Electrophysiology

Female mice (*n* = 10–11/treatment, 31 total) were sacrificed for electrophysiology and hippocampal slices prepared as described previously (18). The uteri of female mice were dissected and weighed prior to LTP assessments, confirming that none of the subjects were in estrus. Mice were anesthetized, decapitated, and the brains rapidly removed into ice-cold, oxygenated dissection medium containing (in mmol/L): 124 NaCl, 3 KCl, 1.25 KH2PO4, 5 MgSO_4_, 0 CaCl_2_, 26 NaHCO_3_, and 10 glucose. Hippocampal slices (340 μm, coronal) were cut from a vibratome (Leica, Model:VT1000S) before transfer to an interface recording containing prewarmed (31 ± 10°C) artificial cerebrospinal fluid composed of (in mmol/L): 124 NaCl, 3 KCl, 1.25 KH2PO_4_, 1.5 MgSO_4_, 2.5 CaCl_2_, 26 NaHCO_3_, and 10 glucose. Slices were perfused continuously at a rate of 1.75–2 mL/minute while the surface of the slices was exposed to warm, humidified 95% O_2_/5% CO_2_. Recordings began following at least 2 hours of incubation.

Field excitatory postsynaptic potentials (fEPSP) were recorded from CA1b stratum radiatum apical dendrites using a glass pipette filled with 2 mol/L NaCl (2–3 MΩ) in response to orthodromic stimulation (twisted nichrome wire, 65 μm diameter) of Schaffer collateral-commissural projections in CA1 stratum radiatum. Pulses were administered 0.05 Hz using a current that elicited a 50% maximal spike-free response. After maintaining a stable baseline (20 minutes), LTP was induced by delivering 5 “theta” bursts, with each burst consisting of four pulses at 100 Hz separated by 200 ms (i.e., theta burst stimulation or TBS). The stimulation intensity was not increased during TBS. Data were collected and digitized by NAC 2.0 (Neurodata Acquisition System, Theta Burst Corp.) and stored on a disk.

Data in the text are presented as means ± SD, while in the figures as mean ± SEM. The fEPSP slope was measured at 10%–90% fall of the slope and data in figures on LTP were normalized to the last 20 minutes of baseline. Electrophysiological measures were analyzed using a one-way ANOVA.

#### Immunofluorescence Imaging

Two 30-μm-thick sections per brain were selected from the ventral hippocampus and the medial prefrontal cortex (mPFC), roughly 300–400 μm apart. Tissues were washed and permeabilized using 0.1% triton in TBS and blocked using 10% goat serum prior to overnight incubation with the following primary antibodies: cluster of differentiation 68 (CD68; 1:500, Bio-Rad), homer scaffold protein 1a (Homer1a; 1:500, Synaptic Systems), Bassoon (BSN; 1:500, Neuromab), Synaptophysin (Syn; 1:500, Sigma), Toll-like receptor 4 (TLR4; 1:500, Novus). Tissues were incubated for 1 hour at room temperature with the following secondary antibodies: Donkey anti-rabbit 488 (1:1,000, Invitrogen), goat anti-mouse 647 (1:1,000, Abcam), and goat anti-rabbit 555 (1:1,000, Invitrogen), before counterstaining with DAPI and being slide mounted. Homer1a and BSN were imaged at 63 × using a ZEISS ELYRA7 with Lattice SIM and postprocessed in Zen and quantified using IMARIS software. CD68 and Syn were imaged on a Nikon Ti2 microscope at 40× magnification.

#### IMARIS Three-dimensional Rendering

All Z-stack images were imported into Imaris version 9.7.0 and deconvoluted using an adaptive, theoretical PSF batch processing. Deconvoluted images were then processed for spot analysis for CD68 and Synaptophysin. To evaluate the mature synaptic binding, Homer1a and BSN super-resolution images were analyzed using spot analysis that confirmed any spot larger than 180 nm, but no larger than 300 nm, as positive. A spot-to-spot analysis was performed to only include Homer1a and BSN spots that were within 180 nm of each other, confirming that the spots were touching and interlocked.

#### ELISA

Immediately after fresh dissection, hippocampal tissue from male animals was flash frozen and stored at −80°C. Tissues were lysed in RIPA buffer and supernatant prepared for ELISA testing. Protein concentrations of lysates were determined using a Bradford assay (Bio-Rad). A cytokine panel ELISA kit (BioLegend LEGENDPLEX) was used to detect IL1β, TNFα, and IL1α from lysates. Results are presented as fluorescent intensity/μg protein and normalized to controls.

### Statistical Analysis

All statistical analysis was performed in Prism (Graphpad Software Inc, version 5.04). For all endpoints, averages of individual animal replicates were used to calculate group interactions using a one-way ANOVA except for fear extinction group analysis where a two-way ANOVA was performed. Upon significant results, Bonferroni *post hoc* testing was performed to determine statistical significance. For behavioral testing, outliers were removed from the statistical analysis. These outliers are defined as scoring outside 2 standard deviations of the mean. Data are presented as mean ± SEM. Values of *P* ≤ 0.05 were considered statistically significant.

### Data Availability

All data reported in this study were generated by the authors. Raw data (video and Prism files) are available on request from the corresponding author.

## Results

### FLASH-RT Does Not Elicit Cognitive Impairments

To date, the long-term impact of hypofractionated FLASH-RT on neurocognitive function has not been reported using an extensive battery of behavioral testing over protracted after irradiation times. While past reports have documented cognitive sparing after single dose exposures or in tumor-bearing mice with a single task (6, 8, 9, 19), it was uncertain whether such benefits would manifest across multiple tasks under the current dosing regimen and between the sexes. Here we also implemented a more rigorous cross-species relevant (meaning that performance metrics share a commonality between rodents and humans) task, namely the OUL (20). The OUL task can be used to evaluate whether/how irradiation interferes with prior associative recognition memories, proving a more rigorous assessment of how animals respond to increasing cognitive load.

**FIGURE 2:**
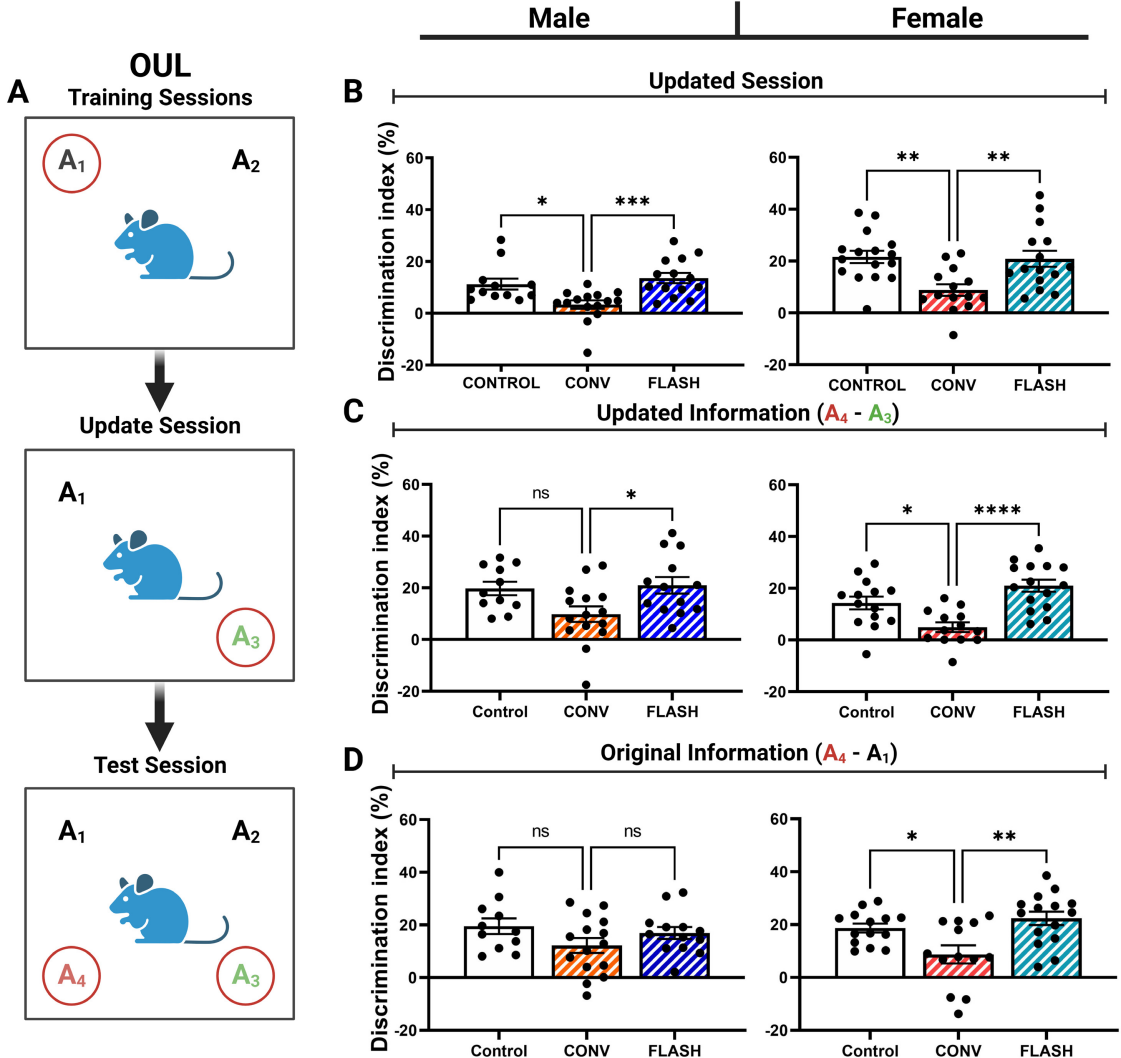
Mice exposed to FLASH-RT performed similar to controls in hippocampal-dependent learning and memory tests OUL while CONV-RT mice did not. **A,** Objects in updated locations testing experimental design. **B,** Update session behavior. At 4 months post-irradiation, FLASH and control mice showed preference for the novel toy and location in both males (left) and females (right) while CONV did not. **C,** Updated information test session. CONV irradiated female mice failed to learn the updated novel (A_4_) object over its predecessor (A_3_) when compared with FLASH and control mice. CONV irradiated male mice performed significantly worse than FLASH mice. **D,** Original information test session. CONV irradiated female mice were unable to differentiate between the updated novel location (A_4_) and the original location (A_1_) while FLASH and control performed similarly. No significant changes in male mice were observed. All data were analyzed using a one-way ANOVA followed by Bonferroni multiple comparison test (*n* = 11–16/sex/treatment). *, *P* ≤ 0.05; **, *P* ≤ 0.01; ns, no significance.

Our behavioral battery was started by assessing performance of male and female mice on the OUL task. A representative image of this test is presented in Fig. 2A. Days 1–3 involved training mice to two identical objects and location, while on day 4 mice were tested on their updated location memory. Control and FLASH irradiated male mice recognized the A_3_ location as novel while CONV irradiated mice did not (Fig. 2B; one-way ANOVA: F_(2,38)_ = 8.616, *P* = 0.0008). These data were corroborated in female mice (Fig. 2B; one-way ANOVA: F_(2,42)_ = 7.336, *P* = 0.0019). Following the updating session, mice performed in the testing session (day 4) where exploration of the toy in a novel location was compared with updated information memory (A4 vs. A3) and the original fixed toy (A4 vs. A1). Male mice exposed to FLASH-RT performed similar to controls, and exhibited DI values that were significantly higher than CONV irradiated male mice (Fig. 2C; one-way ANOVA: F_(2,36)_ = 40.448, *P* = 0.0188). Female mice exhibited similar but more significant differences than males after exposure to FLASH-RT (Fig. 2C; one-way ANOVA: F_(2,39)_ = 12.57, *P* < 0.0001). When comparing novel location with original information (A4 vs. A1), male mice exhibited similar albeit nonsignificant trends (Fig. 2D; one-way ANOVA: F_(2,36)_ = 1.929, *P* = 0.16); however, FLASH irradiated female mice performed the same as controls while CONV irradiated female mice performed significantly worse (Fig. 2D; one-way ANOVA: F_(2,39)_ = 7.236, *P* = 0.0021).

**FIGURE 3:**
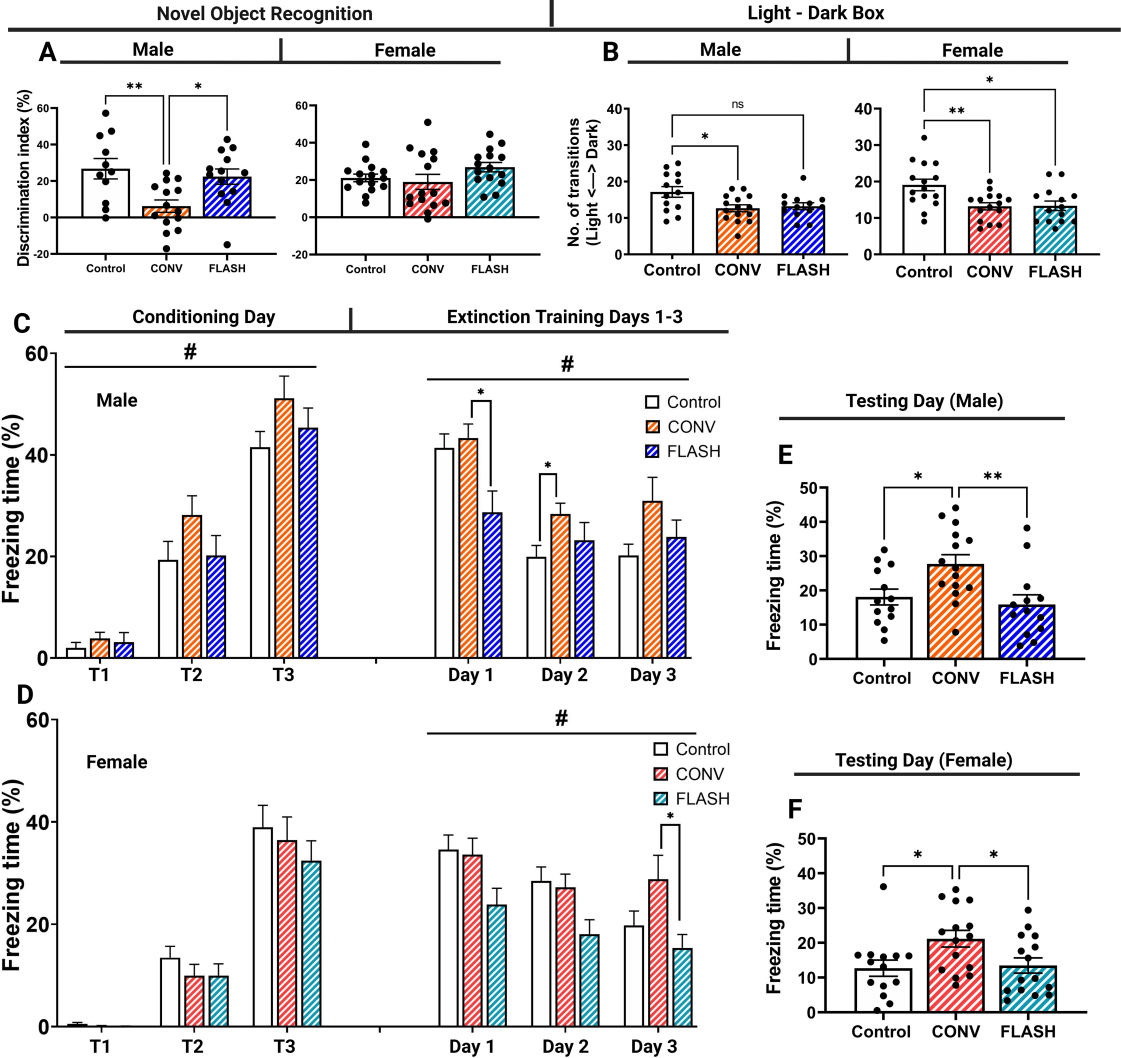
Mice exposed to FLASH-RT performed similar to controls in the NOR, LDB, and FE tests. **A,** NOR testing. Male mice exposed to FLASH-RT performed similar to controls, while CONV-RT were unable to differentiate between the familiar and novel object. Female mice exposed to FLASH-RT and CONV-RT exhibited no significant difference between controls. **B,** Measurement of transition between light and dark environments in LDB testing. Male and female mice exposed to CONV-RT performed significantly worse than controls. Male FLASH-RT mice were not significantly different than controls; however, female mice did not transition between arenas as controls did. **C** and **D**, Fear extinction training and extinction days. Exposure to CONV-RT caused male mice to exhibit increased freezing during training, while this was not observed in FLASH-RT or females. Exposure to CONV-RT also inhibited mice ability disassociate the tone/shock pairing as well as FLASH and controls in males and females. Group effects (#) were found in training days indicate that CONV irradiated animals exhibited increase freezing behavior. **E** and **F,** Fear extinction testing. FLASH and control mice greatly reduced their tone/shock associations while CONV male (left) and female (right) mice did not. Data were analyzed using a one-way or two-way ANOVA followed by Bonferroni multiple comparison test (*n* = 11–15/sex/treatment). *, *P* ≤ 0.05; **, *P* ≤ 0.01; #, *P* ≤ 0.05; ns, no significance.

Following OUL testing, mice were analyzed on the NOR task to evaluate episodic memory. Male mice exposed to FLASH-RT were indistinguishable from control mice while CONV irradiated mice performed significantly worse than either (Fig. 3A; one-way ANOVA: F_(2,35)_ = 6.403, *P* = 0.0043). Female mice exposed to FLASH-RT performed similar to controls; however, the CONV-RT cohort did not show a decrement after irradiation (Fig. 3A; one-way ANOVA: F_(2,42)_ = 2.922, *P* = 0.0648).

After completion of the NOR test, mice were evaluated in the LDB arena. Anxiety is assessed by the number of transitions made between the light and dark areas. Results from this test indicate that control mice performed better than CONV-RT and FLASH-RT in both males (Fig. 3B; one-way ANOVA: F_(2,38)_ = 4.603, *P* = 0.0162) and females (Fig. 3B; one-way ANOVA: F_(2,41)_ = 6.548, *P* = 0.0034).

The behavioral battery was concluded by assessing freezing behavior of mice in the fear extinction test. Fear extinction refers to the dissociation of a learned response to a prior adverse effect. Both male and female mice learned to associate a tone and mild foot shock during the conditioning phase of the trial. During extinction training (days 2–4), mice were repeatedly exposed to the tone in a new environment and freezing behavior was measured, providing time for mice of both sexes and treatment to dissociate the learned response in a new hippocampal-dependent context. In males, a group effect was found during training (Fig 3C; two-way ANOVA: F_(2,40)_ = 3.376, *P* = 0.0442) and extinction training (Fig 3C; two-way ANOVA: F_(2,40)_ = 4.121, *P* = 0.0236) indicating that CONV irradiation caused animals to increase freezing behavior. A group effect was also found in female animals during the extinction training phase (Fig 3D; two-way ANOVA: F_(2,45)_ = 3.287, *P* = 0.0465) indicating that CONV irradiated animals were unable to reduce freezing behavior when shock stimulus was removed. On the final day of testing, mice are returned to the original environment where they received a mild foot shock followed by a tone. CONV irradiated mice exhibited higher levels of freezing than control or FLASH in both males (Fig 3E; one-way ANOVA: F_(2,38)_ = 5.982, *P* = 0.0055) and females (Fig 3F; one-way ANOVA: F_(2,41)_ = 4.145, *P* = 0.0229), indicating that FLASH-RT preserved extinction memory in both sexes of mice.

### Electrophysiological Evaluation Reveals the Capability of FLASH-RT to Preserve LTP

Electrophysiology provides for direct and functional measures of neurotransmission, which can clearly impact behavioral outcomes, and to date, no such measurements have been recorded from the brains of FLASH irradiated mice. Because of the nature of these experiments, prior exposure to mild electrical shock (FE task) could confound such measurements, which necessitated the analysis of a separate cohort of mice (female) subjected to the same hypofractionated regimen. As LTP provides a validated method for assessing synaptic plasticity (21), we hypothesized that this measure of activity-dependent synaptic connections between interconnected hippocampal circuitry might be preserved after FLASH-RT versus CONV-RT at protracted timepoints.

**FIGURE 4:**
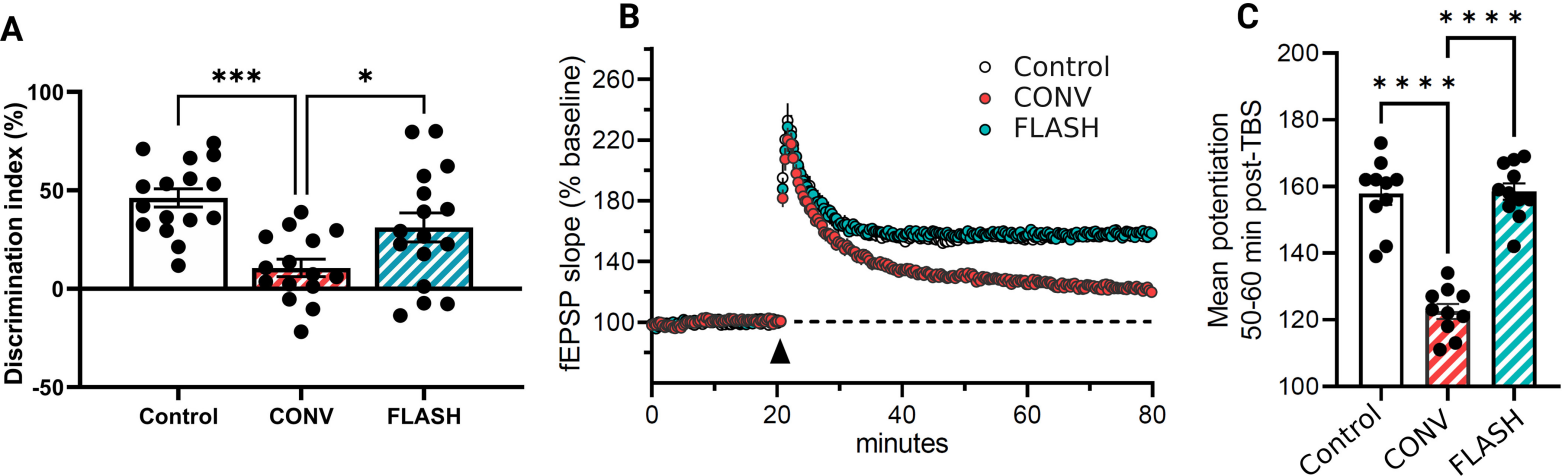
FLASH irradiation protects against reductions in LTP after CONV irradiation, 6 months after irradiation. **A,** Extended novel object recognition testing, 4 months after irradiation. Female mice that exposed to FLASH-RT performed significantly better than those who received CONV-RT (*n* = 15–16/treatment). **B,** TBS applied to the Schaffer collaterals produced a robust increase in fEPSP slope (as percent of baseline) in control and FLASH irradiated female mice but reduced in CONV mice 6 months after exposure. **C,** Levels of potentiation in the fEPSP slope maintained 1 hour after TBS was reduced significantly in the hippocampus of CONV-RT mice, but not in control or FLASH irradiated mice. Data were analyzed using a one-way ANOVA followed by Bonferroni multiple comparison test (*n* = 10–11/treatment). *, *P* ≤ 0.05: ***, *P* ≤ 0.001; ****, *P* ≤ 0.0001.

To test this, we irradiated a cohort of female mice at 10 weeks of age and tested them 4 months later on an extended (1 day between exploration and testing) NOR task to confirm neurocognitive sparing before LTP assessment. Mice exposed to FLASH-RT performed similar to controls, while CONV irradiated mice were unable to differentiate between the novel and familiar toy (Fig 4A; one-way ANOVA: F_(2,44)_ = 9.711, *P* = 0.0003). Interestingly, and opposed to the short-term version of this assay on female mice (Fig. 3A), the extended NOR assay was able to validate the FLASH effect in this cohort. Following NOR testing, female animals were subjected to the measurement of hippocampal LTP along the Schaffer collaterals.

TBS applied to the Schaffer collaterals produced a rapid and robust increase in LTP, quantified as the relative change in the slope of evoked fEPSPs generated by CA1 apical dendrites (Fig. 4B). Following the TBS, the fEPSP slope gradually decayed to more stable levels of potentiation for all cohorts. Notably, mean potentiation levels in the fEPSP slope maintained at 1 hour after TBS were reduced significantly in the hippocampus following CONV-RT, but not in control or following FLASH-RT (Fig. 4C; one-way ANOVA: F_(2,28)_ = 56.99, *P* < 0.0.0001). Moreover, the fact that these data were collected at protracted after irradiation times (6 months), suggests that CONV-RT elicits a relative permanent inhibition of LTP, whereas unirradiated controls and FLASH irradiated mice were remarkably, statistically indistinguishable. Such robust functional readouts demonstrate that FLASH-RT can protect the normal tissue structure function relationships of the mouse brain such that synaptic plasticity that underlies critical learning and memory processes can be preserved.

### FLASH Irradiation Preserves Synaptic Density in the Hippocampus and mPFC

**FIGURE 5:**
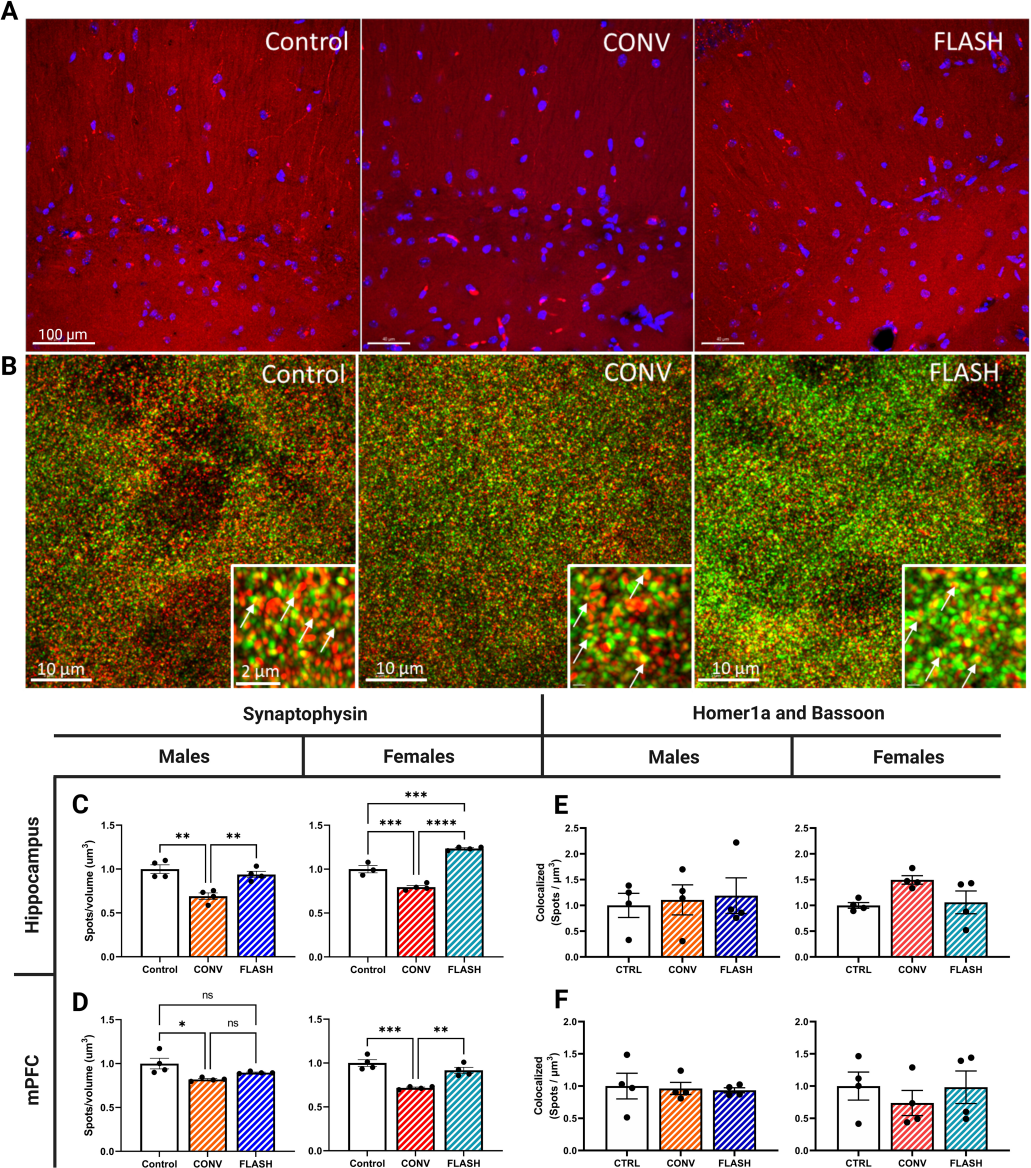
FLASH irradiation protects synaptic density and spine morphology, 6 months after irradiation. **A,** Representative images of synaptophysin (red), DAPI (blue; Scale bar = 100 μm). **B,** Representative image of Homer1a (red)/Bassoon (green), respectively (Scale bar = 10 μm and 2 μm in the zoomed image). **C** and **D**, Quantification of synaptic density using synaptophysin found that FLASH did not induce dendritic disruptions that were observed in mice exposed to CONV-RT in both the Hippocampus and mPFC. **E** and **F**, Quantification of Homer1a and Bassoon spots within 120 nm of each other. Male and female mice exhibited no differences between presynaptic and postsynaptic binding after FLASH or CONV irradiation in the hippocampus or mPFC. All data were analyzed using a one-way ANOVA followed by Bonferroni multiple comparison test (*n* = 4/sex/treatment, two sections analyzed/region/animal). *, *P* ≤ 0.05; **, *P* ≤ 0.01; ***, *P* ≤ 0.001; ****, *P* ≤ 0.0001; ns, no significance.

To further evaluate the neurobiologic consequences of dose-rate modulation in the irradiated rodent brain, we analyzed potential changes in select synaptic markers (Fig. 5). Synaptophysin is a well-described integral membrane marker localized to presynaptic dendrites that has been used to measure synaptic plasticity (22) and neuronal architecture (23). Expression of the major synaptic vesicle protein synaptophysin was evaluated to quantify presynaptic vessel density in both the hippocampus and mPFC (Fig. 5A). A significant decrease in presynaptic synaptophysin density after CONV-RT was observed in the hippocampus that was not found after FLASH-RT, 6 months after irradiation in both males (Fig. 5C; one-way ANOVA: F_(2,9)_ = 15.82, *P* = 0.0011) and females (Fig. 5C; one-way ANOVA: F_(2,8)_ = 98.95, *P* = 0.0001). These data were further corroborated in the mPFC finding similar significant differences in females (Fig. 5D; one-way ANOVA: F_(2,9)_ = 23.55, *P* = 0.0003), but not in FLASH irradiated males (Fig. 5D; one-way ANOVA: F_(2,9)_ = 6.458, *P* = 0.0182). Taken together, these data indicate that FLASH-RT spared synaptophysin density in two distinct regions of the brain, in stark contrast to CONV-RT.

In our previous studies, we have shown that FLASH-RT preserved spine density and dendritic complexity after irradiation (9). To further scrutinize synaptic connections, we used an ELYRA7 super-resolution microscope to evaluate presynaptic and postsynaptic connectivity (Fig. 5B). Previous studies evaluating synaptic bouton suggested that an analysis of the juxtaposition of presynaptic BSN and postsynaptic homer scaffold protein 1a (Homer1a) puncta would provide a robust analysis of synaptic connections (24). Our results indicate that no significant changes were found after either irradiation modality in either the male (Fig. 5E; one-way ANOVA: F_(2,9)_ = 0.1032, *P* = 0.9030) or female (Fig. 5E; one-way ANOVA: F_(2,9)_ = 3.744, *P* = 0.0656) hippocampus, or the male (Fig. 5F; one-way ANOVA: F_(2,9)_ = 0.07531, *P* = 0.928) or female (Fig. 5F; one-way ANOVA: F_(2,9)_ = 0.4298, *P* = 0.6633) mPFC.

### FLASH-RT Does Not Elicit an Inflammatory Response After Hypofractionation at a Protracted Time

**FIGURE 6:**
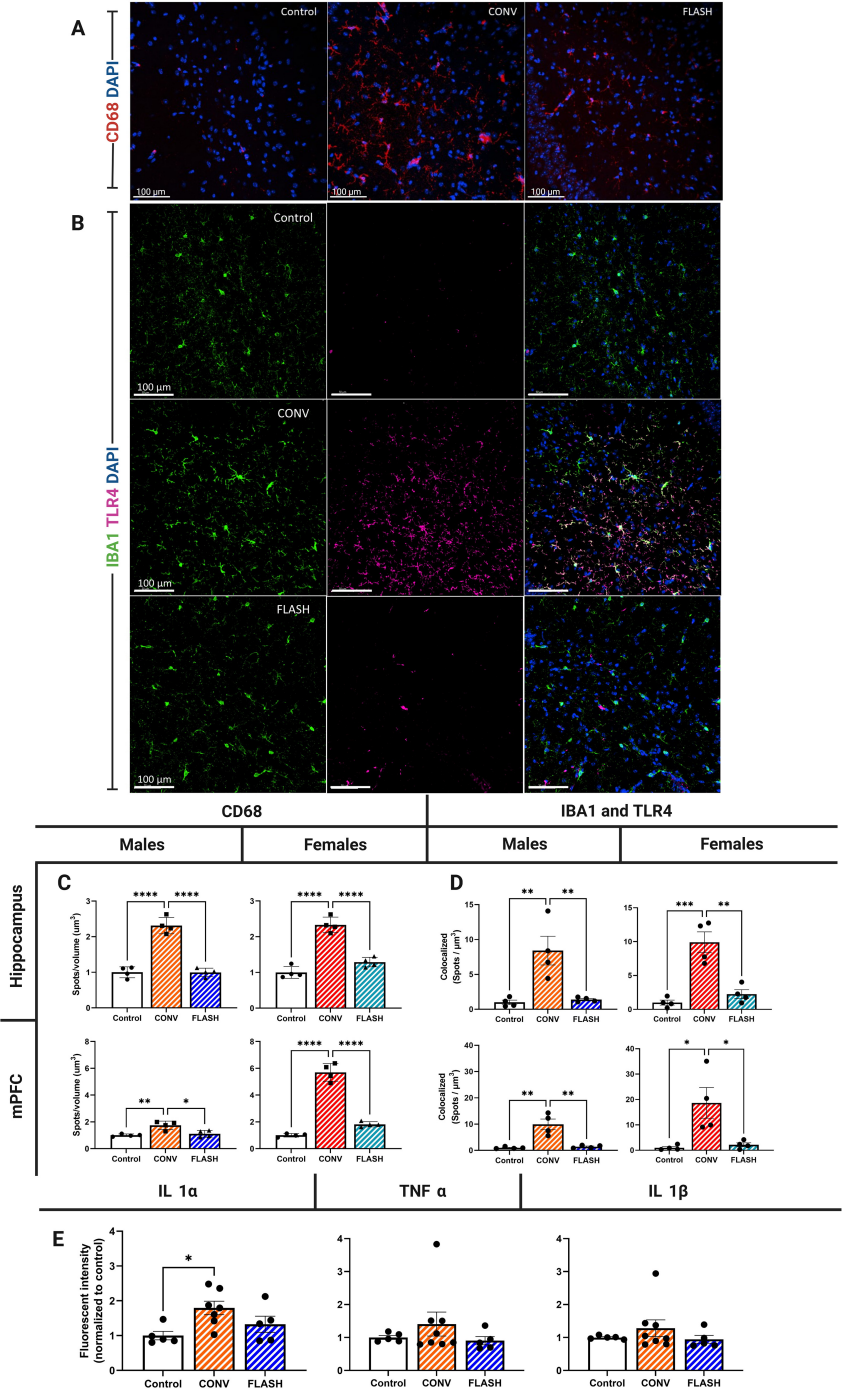
FLASH irradiation protected against prolonged inflammation found in CONV mice, 6 months after irradiation. Representative images of reactive microglia CD68 (red) and DAPI (blue) in the male mouse hippocampus (**A**) and representative images of IBA1 (green), TLR4 (red), and DAPI (blue; Scale bar = 100 μm; **B**). **C,** Quantification of CD68 immunofluorescence in the hippocampus and medial prefrontal cortex. Male (left) and female (right) mice exposed to FLASH-RT exhibit no significant change in CD68 expression while CONV mice expressed a neuroinflammatory response. **D,** Quantification of IBA1 and TLR4 colocalization in the hippocampus and medial prefrontal cortex. Male and female mice exhibited decreased levels of the neuroinflammatory mediator TLR4 when compared with CONV irradiation. **E,** Inflammatory cytokines measured using ELISA. IL1α exhibited elevated expression after CONV-RT exposure when compared with controls while FLASH induced no changes. No significant changes were observed in TNFα or IL1β. All data were analyzed using a one-way ANOVA followed by Bonferroni multiple comparison test (*n* = 4/sex/treatment, two sections analyzed/region/animal). *, *P* ≤ 0.05; **, *P* ≤ 0.01; ****, *P* ≤ 0.0001.

Previous work has documented a robust inflammatory response associated with cognitive impairments occurring after radiotherapy (25). To assess whether FLASH-RT induced long-lasting neuroinflammation, measurements of reactive microglia (CD68; Fig. 6A) and quantification of IBA1 (microglia) and TLR4 colocalization (Fig. 6B) were analyzed using immunofluorescent staining. Data indicated that a robust inflammatory response was found in the hippocampus of mice exposed to CONV-RT; however, FLASH-RT animal expressed levels similar to control in males (Fig. 6C; one-way ANOVA: F_(2,9)_ = 75.49, *P* < 0.0001) and females (Fig. 6C; one-way ANOVA: F_(2,9)_ = 63.31, *P* < 0.0001). These findings were corroborated in the mPFC in males (Fig. 6C; one-way ANOVA: F_(2,9)_ = 11.21, *P* = 0.0036); however, FLASH irradiated females also expressed higher levels of inflammation that controls, though significantly less than CONV (Fig. 6C; one-way ANOVA: F_(2,9)_ = 153.7, *P* < 0.0001).

Toll-like receptor 4 (TLR4) is known as a mediator of inflammation which can trigger proinflammatory cytokines (26). Data from IBA1 and TLR4 colocalization analysis found that CONV-RT induced an inflammatory response while FLASH-RT protected the brain, similar to our previous findings of reactive microglia (CD68) in both the male (Fig. 6D; one-way ANOVA: F_(2,9)_ = 11.96, *P* = 0.0029) and female (Fig. 6D; one-way ANOVA: F_(2,9)_ = 23.91, *P* = 0.0003) hippocampus. These findings were corroborated within the mPFC in both males (Fig. 6D; one-way ANOVA: F_(2,9)_ = 18.16, *P* = 0.0007) and females (Fig. 6D; one-way ANOVA: F_(2,9)_ = 7.804, *P* = 0.0108). Because TLR4 is known to induce proinflammatory cytokines we assessed tissues of male mice using ELISA. Our results indicated that CONV-RT elevated levels of IL1α when compared with controls while FLASH-RT caused no change (Fig. 6E; one-way ANOVA: F_(2,14)_ = 4.554, *P* = 0.03). While similar trends of elevated IL1β and TNFα after CONV-RT were observed, these did not reach significance. These results are consistent with previous findings that coevaluated neuroinflammation with neurologic damage and cognitive impairment (6, 9).

## Discussion

Here we describe the first comprehensive and long-term assessment of critical functional, cellular, and molecular outcomes in the hypofractionated FLASH irradiated mouse brain. The fact that control and FLASH cohorts exhibited outcomes that were statistically similar (if not indistinguishable) across such a varied series of endpoints is remarkable, considering the protracted times of follow-up. In nearly every instance, CONV-RT led to significant disruptions in each endpoint measured that was not observed after FLASH-RT. Neurocognitive benefits tracked with the preservation of synaptic plasticity and integrity through multiple measures that were coincident with reductions in neuroinflammation.

To safely implement FLASH-RT into the clinic the convergence of multiple expertise and continued research is needed. To that end, we have focused on delivering a high total fractionated dose to the brain, close to the standard of care currently used for the treatment of brain metastasis (27, 28) in efforts to establish a link between critical functional outcomes and key cellular, molecular, and structural mediators of neurotransmission. Our initial focus was to critically evaluate the long-term capability of hypofractionated FLASH-RT to spare neurocognition using an expanded behavioral battery by the inclusion of a cross-species relevant OUL task and an extended (24 hours) NOR task in tumor-free animals. It was noteworthy, especially after a total dose of 30 Gy, that data derived from four distinct tasks conducted 4–6 months after irradiation, routinely showed that FLASH tracked control cohorts and did not exhibit the significant deficits observed after CONV-RT. Interesting too was that the cognitive benefits of FLASH-RT extended (for the most part) across both sexes, and in no instance was FLASH observed to be more deleterious than CONV-RT. FLASH may not elicit the same level of radiolytic change to preexisting structural elements critical for synaptic transmission. Whether reduced damage to certain normal tissue targets favors faster tissue repair or remodeling cannot be formally ruled out; however, we have found similar normal tissue sparing in the brain (albeit after different irradiation regimen) from 1–6 months after exposure. In the end, whatever FLASH damage was produced appears to be below the threshold required to elicit or manifest functional change in the central nervous system (CNS). Such results portend favorable new treatment options for those suffering with brain tumors, where the use of hypofractionated radiosurgery or SBRT-FLASH (brain metastasis) or whole brain FLASH-RT (unresectable glioma, palliative care) may help remediate mid- and longer-term neurocognitive complications.

The fact that indices of learning and memory remained relatively intact after a 30 Gy hypofractionated dose of FLASH-RT points to the preservation of certain synaptic elements involved in neurotransmission. In this regard, electrophysiological assessments provide direct measures of electrical activity within the brain, and long-term potentiation has remained a reliable standard in the field for assessing synaptic plasticity (29, 30). Past work from our lab (albeit using distinctly different irradiation paradigms) has found that paired cell recordings are able to uncover subtle yet significant radiation-induced changes in the excitability profiles of principal cells in select cortical and hippocampus subfields (31, 32). These changes become more significant when excitability is assessed over larger networks involving multiple synapses, suggesting that LTP measures within the hippocampal CA1 might reveal dose rate–dependent effects (31, 33). Indeed, FLASH-RT preserved hippocampal LTP identically compared with control cohorts, whereas CONV-RT inhibited LTP significantly. Interestingly, data also suggest that LTP may serve as a biomarker of the FLASH effect, and ongoing studies will confirm the time course of dose rate–dependent changes in LTP at earlier after irradiation times. In addition, preservation (FLASH) and inhibition (CONV) of LTP appear permanent after irradiation, but additional studies may identify synaptic substrates able to potentiate or reverse LTP shortly after TBS, in efforts to further evaluate the plasticity of molecular events involved in consolidation of LTP after irradiation. The fact that FLASH-RT preserved this measure of synaptic function corroborates our cognitive data and suggests that FLASH-RT does not perturb the underlying circuit firing along a pathway (Schaffer collaterals) in a hippocampal region evaluated in our cognitive testing. These data suggest that even after hypofractionated FLASH-RT, synaptic functional integrity and neurotransmission can be spared, effects that are clearly perturbed after CONV irradiation.

To assess more formally what components of synaptic architecture might be preserved or disrupted after FLASH- or CONV-RT, we utilized IHC to quantify levels of the presynaptic marker synaptophysin in the hippocampus and mPFC. The capability of FLASH-RT to preserve the density of presynaptic synaptophysin vesicles, which was compromised significantly after CONV-RT suggest a potential underlying mechanism for the preservation of synaptic plasticity assessed by out LTP measurements. To provide a higher resolution analysis of synaptic structures, we utilized super-resolution microscopy to quantify the extent of colocalization between presynaptic and postsynaptic bouton (24). The close association (≤100 nm) of Bassoon in the presynaptic active zone and Homer-1 in the postsynaptic density presumes “tight binding” and provides an indication of whether the structural interaction between these integral synaptic scaffolding proteins was maintained (or not) after FLASH-RT. Colocalization data indicated that the association between Bassoon and Homer-1 in the hippocampus and medial prefrontal cortex was not changed significantly by irradiation whereas LTP was spared by FLASH-RT only. These results suggest that FLASH preserves synaptic function and the release of neuromediators required for neurocognitive function.

While the primary mechanism by which FLASH limits toxicity in the brain remains difficult to pinpoint, it was not the aim of this study, which focused on later biological events. Our data have provided considerable evidence linking the beneficial effects of FLASH-RT from synapse to cognition. Other publications have however, focused on possible primary events in efforts to rationalize how FLASH might spare normal tissue toxicity which have invoked differential free radical cascades and reduced secondary yields of ROS after FLASH versus CONV (1, 9), in addition to the striking absence lipid peroxidation after FLASH versus CONV (34). These factors likely limit neuroinflammation, in addition to other mechanisms as proposed in our recent review (5). Here we can speculate that a “certain as yet unidentified” preexisting structural motif may define a target unique to normal tissue that is not as susceptible to radiolytic change. Whether this target is either absent or altered in tumors that renders them equally susceptible to dose-rate modulation is uncertain, but based on all available data to date, the FLASH effect likely involves multiple complex responses that are distinct between normal tissue and tumors (5).

Microglia play pleiotropic roles in maintaining the health of the CNS, effects that depend on the specific context (age, disease, endo/exogenous stressor) in which they exist. As the innate macrophages of the CNS, they participate in gliovascular and synaptic remodeling through process motility, secretion of soluble factors and their capacity for phagocytosis (35). In the irradiated CNS, microglia likely operate though a “sensome” that facilitates their capability to transition between dynamic reactive states able to survey, detect, and quickly respond to changes in their local environment (35). This enables multifunctionality, where microglia can preserve synaptic and vascular integrity (FLASH) or enact opposite responses (CONV) depending on the local cues in which they respond (36–38). Precisely how these local cues differ in response to dose-rate modulation is uncertain at present but might involve different free radical cascades, as suggested above. Nonetheless, evidence clearly indicates that the sustained microglial response is sensitive to dose rate–induced changes, where the preservation of a more “normal” unirradiated homeostatic state is more readily achieved when a toxic dose is delivered at FLASH dose rates. Indeed, past work has delineated a variety of important roles microglia have in directly mediating the radiation response of the CNS (25, 39), and how they can modulate information processing important for cognition by potentiating or suppressing inflammation in the brain (40, 41). In response to CONV-RT and other higher linear energy transfer (LET) modalities, reactive microglia have been linked to impaired cognition through the complement signaling cascade (42), reactive astrogliosis and microgliosis that elevate inflammatory cytokines (9). Many of these proinflammatory signatures can be attenuated by microglial depletion (25, 43), inhibition of adenosine kinase (44) and the HMGB1/TLR4 signaling axis (45) or in the case of past (6, 9, 10) and present findings FLASH-RT. The marked capability of FLASH-RT to suppress (if not prevent) persistent elevations in microglial activation after a high-dose, hypofractionated regimen point to one of the more significant outcomes of this new cancer treatment modality that should hasten clinical translation.

Using a well-characterized FLASH beam (eRT6, Oriatron), this work now provides a proof of concept that hypofractionated FLASH irradiation is beneficial over protracted postexposure times. While this device is clearly not suitable for clinical radiotherapy of brain tumors, proton FLASH does currently have the beam characteristics more favorable for immediate to mid-term clinical translation. Notwithstanding *in vivo* validation of normal tissue sparing in the brain with pencil beam scanning of larger volumes treated with proton FLASH, the development of very high energy electron and photon FLASH beams may provide suitable solutions in the future. On the topic of mechanism, a topic of intense interest in the field, we can pro-offer two ideas. As alluded to above, we suspect that normal cells have certain preexiting structural elements that are resistant to radiolytic change at ultra-high dose rates, but one might simply surmise that FLASH and CONV kill tumors the same way. In the second idea, FLASH may induce a metabolic switch in normal cells that promotes a state of quiescence, one that normal tissues can tolerate but tumors cannot. Reduced transcriptional, translational stress and lower macromolecular synthesis may alleviate normal tissue toxicities but may be more consequential to tumors that are more reliant on such processes for growth and survival. We have discussed many of these possibilities among others in further detail in a recent review (5).

As the landscape of modern radiotherapy continues to evolve and improve, so too have patient outcomes. Technological and biological advancements have ushered in a new era of stereotactic conformality that can be coupled with more tumor selective agents that are clearly extending overall survival for nearly every cancer, especially those diagnosed before oligometastatic dissemination (46). The challenge of targeting malignant subpopulations of cancer cells within our most structurally complex and important organ cannot be overstated, and while the eventual eradication of brain cancer remains a challenge, it is perhaps the target organ that stands to benefit the most from FLASH-RT. While neurosurgery remains the standard, the capability of ionizing radiation to noninvasively penetrate the protective structures of the brain provides FLASH-RT coupled with SBRT and radiosurgery to pursue curative intent while maintaining acceptable long-term normal tissue toxicities for the benefit of patients with brain tumors.
